# A Prediction Nomogram Combining Epworth Sleepiness Scale and Other Clinical Parameters to Predict Obstructive Sleep Apnea in Patients with Hypertension

**DOI:** 10.1155/2022/3861905

**Published:** 2022-08-05

**Authors:** Lin Wang, Dongsheng Sun, Jianhong Xie, Li Zhang, Dibo Lao, Shaokun Xu

**Affiliations:** Geriatric Medicine Center, Department of Geriatric Medicine, Zhejiang Provincial People's Hospital (Affiliated People's Hospital, Hangzhou Medical College), Hangzhou, Zhejiang 310014, China

## Abstract

**Background:**

Obstructive sleep apnea (OSA) is common in patients with hypertension. Nonetheless, OSA is underdiagnosed despite considerable evidence of the association between OSA and adverse health outcomes. This study developed and validated a clinical nomogram to predict OSA in patients with hypertension based on the Epworth Sleepiness Scale (ESS) score and OSA-related parameters.

**Methods:**

A total of 347 hypertensive patients with suspected OSA were retrospectively enrolled and randomly assigned to a training set and a validation set at 70 : 30 (*N* = 242/N = 105) ratio. OSA was diagnosed through sleep monitoring and was defined as an apnea-hypopnea index ≥5 events/h. Using the least absolute shrinkage and selection operator regression model, we identified potential predictors of OSA and constructed a nomogram model in the training set. The predictive performance of the nomogram was assessed and validated by discrimination and calibration. The nomogram was also compared with ESS scores according to decision curve analysis (DCA), integrated discrimination index (IDI), and net reclassification index (NRI).

**Results:**

ESS scores, body mass index, neck circumference, snoring, and observed apnea predicted OSA are considered. The nomogram showed similar discrimination between the training set (AUC: 0.799, 95% CI: 0.743–0.847) and validation set (AUC: 0.766, 95% CI: 0.673–0.843) and good calibration in the training (*P*=0.925 > 0.05) and validation (*P*=0.906 > 0.05) sets. Compared with the predictive value of the ESS, the nomogram was clinically useful and significantly improved reclassification accuracy (NRI: 0.552, 95% CI: 0.282–0.822, *P* < 0.001; IDI: 0.088, 95% CI: 0.045–0.133, *P* < 0.001) at a probability threshold of >42%.

**Conclusions:**

We developed a novel OSA prediction nomogram based on ESS scores and OSA-related parameters. This nomogram may help improve clinical decision-making, especially in communities and primary clinics, where polysomnography is unavailable.

## 1. Introduction

Obstructive sleep apnea (OSA) is the most common sleep-disordered breathing (SDB), with a prevalence of 2%–26% in adults [1–3]. OSA increases the risk of hypertension [4] and cardiovascular [5], cerebrovascular [6], and metabolic diseases [7, 8]. In this respect, approximately 30% of patients with primary hypertension and up to 80% of patients with drug-resistant hypertension have OSA [9]. Additionally, OSA increases the risk of hypertension-related morbidities [9]. The prevalence of hypertension is positively correlated with OSA severity [10]. The coexistence and worsening of OSA and hypertension increase the risk of adverse health outcomes [11]. Thus, the early diagnosis and treatment of OSA in patients with hypertension are crucial.

At present, polysomnography (PSG) and portable monitoring (PM) are the gold standards for diagnosing OSA [12, 13]. However, these diagnostic methods are expensive and complex, and their availability is limited. In addition, the waiting time for diagnosis and treatment is long [14]. Late diagnosis delays OSA treatment, increasing the risk of comorbidities. Therefore, a simple and reliable method to identify and screen patients at a high risk of OSA is urgently needed.

Clinical questionnaires and scales (i.e., Epworth Sleepiness Scale [ESS], the STOP-Bang questionnaire [SBQ], and the Berlin questionnaire [BQ]) are used to diagnose OSA in the absence of standard PSG; however, these questionnaires have limitations [15]. Many OSA-related parameters, including body mass index (BMI), neck circumference (NC), gender, and comorbidities, are relevant for predicting OSA [16, 17]. This study constructed and validated a simple-to-use nomogram based on ESS scores and OSA-related parameters to diagnose OSA in patients with hypertension.

## 2. Materials and Methods

### 2.1. Study Subjects

This cross-sectional study used data from 347 hypertensive patients with suspected OSA, who were referred to the Hypertension Clinic of the People's Hospital of Zhejiang Province between January 1, 2019, and December 30, 2020. We excluded patients with hypothyroidism or hyperthyroidism, Cheyne-Stokes respiration, ischemic or hemorrhagic stroke, nasopharyngeal or oropharyngeal diseases (uncontrolled acute tonsillitis, acute otitis media, rhinitis, and sinusitis), missing questionnaires or information, and patients aged <18 and >80 years.

### 2.2. Portable Monitoring

All subjects underwent whole-night sleep monitoring with a PM (Murrysville, America) at home from 10 pm until 6 am the next day, with at least 7 h of recording. PM measured the airflow (nasal/oral thermocouple and a nasal pressure transducer), respiratory effort (thoracic and abdominal), arterial oxygen saturation and pulse rate, snoring time and intensity, and changes in body position.

Apnea was defined as the complete cessation of airflow or a ≥90% decrease in peak thermal sensor excursion for at least 10 s. Hypopnea was defined as a decrease in nasal pressure signal excursion of ≥50% from baseline with a SpO_2_ decrease of >3% from baseline for ≥10 s [18]. The apnea-hypopnea index (AHI) was calculated as the total number of OSA and hypopnea episodes per hour of sleep. Subjects were divided into an OSA group (AHI ≥5 events/h) and a control group (AHI <5 events/h) [18].

### 2.3. Data Collection and Potential Predictors

Demographic characteristics (age, sex, occupation, height, weight, and NC), OSA symptoms (snoring, tiredness, and observed apnea), tea consumption, cigarette consumption, disease history (including cerebrovascular and respiratory diseases), and answers to the ESS questionnaire were collected before PM. BMI was defined as kg/m^2^.

### 2.4. ESS Evaluation

The ESS consists of eight self-rated items and assesses the probability of dozing using a scale ranging from 0 (never) to 3 (high). The sum of each item score yielded a global score (range, 0–24) [19]. ESS, originally designed to assess the risk of daytime sleepiness, can determine the subjective probability of falling asleep in various environments, and is used to detect OSA [20]. This study used the Chinese version of the ESS [21] and considered ESS scores as a continuous variable.

### 2.5. Statistical Analysis

Descriptive analyses were conducted using SPSS version 22.0 for Windows (SPSS Inc., Chicago, IL, USA). Continuous variables were summarized as mean ± standard deviations or medians (interquartile ranges) and were compared using student' *t*-test or Wilcoxon rank-sum test. Categorical variables were expressed as percentages and were compared using Pearson chi-square test.

The derivation and assessment of the nomogram were performed according to the five steps: (1) All subjects were randomly assigned to a training set (to construct the nomogram) and a validation set at a 70 : 30 ratio (*N* = 242/N = 105); (2) Independent potential predictors of OSA in the training set were identified using the least absolute shrinkage and selection operator (LASSO) regression model [22, 23]; (3) The nomogram model based on these predictors was developed by multivariate logistic regression analysis, and the results were presented as odds ratios (ORs), with associated 95% confidence intervals (95% CIs) and corresponding *P*-values [24]; (4) In the training and validation sets, discrimination and calibration of the nomogram model were assessed using the area under the receiver operating characteristic (ROC) curve (AUC) and calibration curve plot, respectively [25]. AUC >0.75 indicated good discrimination of the model. *P* > 0.05 in the calibration curves suggested good consistency between the new model and standard diagnostic criteria; (5) The clinical benefits and utility of the nomogram compared with the ESS were evaluated using the net reclassification index (NRI) [26], integrated discrimination improvement (IDI) [27], and decision curve analysis (DCA) [28].

The LASSO, nomogram, ROC curve, DCA, NRI, IDI, and bootstrap analysis were performed using the packages “glmnet,” “rms,” “ROCR,” “rmda,” “nricens,” and “PredictABEL” in R version 4.0.1, respectively. Statistical significance was set at *P* > 0.05.

## 3. Results

### 3.1. Baseline Characteristics

In total, 347 subjects with a mean age of 46.6 ± 12.4 years were enrolled. Men accounted for 77.8% of the cohort. There were no significant differences in baseline characteristics between the training and validation sets (*P*=0.091 − 0.963) (Table 1).

### 3.2. Selection of Predictors by LASSO Regression

**Figure 1:**
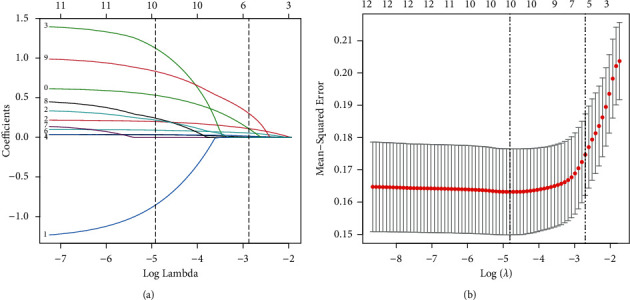
Demographic, anthropometric value, OSA-related signs and symptoms, and ESS score selection using the LASSO binary logistic regression model. (a) Optimal parameter (lambda) selection in the LASSO model used ten fold cross-validation via minimum criteria. The partial likelihood deviance (binomial deviance) curve was plotted versus log(lambda). Dotted vertical lines were drawn at the optimal values by using the minimum criteria and the 1 SE of the minimum criteria (the 1-SE criteria). (b) LASSO coefficient profiles of the 12 features. A coefficient profile plot was produced against the log(lambda) sequence. Vertical line was drawn at the value selected using ten-fold cross-validation, where optimal lambda resulted in five features with nonzero coefficients. LASSO, least absolute shrinkage and selection operator; SE, standard error. OSA, Obstructive sleep apnea; ESS, Epworth sleepiness scale.

Potential predictors of OSA in the training set were identified using the LASSO regression model. Five factors-ESS scores, BMI, NC, snoring, and observed apnea-were significant predictors of OSA (Figures 1(a), 1(b)) and had nonzero coefficients (Lambda.1se = 0.05989) in the 10-fold cross-validation LASSO regression model (Table 2).

### 3.3. Multivariate Logistic Regression Analysis in the Training Set

OSA and the five predictors were considered dependent and independent variables, respectively. The results of multivariate logistic regression analysis showed that ESS scores (per 1 score increase) (OR = 1.24, 95% CI: 1.12–1.38), BMI (OR = 1.06, 95% CI: 0.94–1.20), NC (OR = 1.20, 95% CI: 1.04–1.38), snoring (OR = 2.36, 95% CI: 0.94–5.92), and observed apnea (OR = 1.88, 95% CI: 0.87–4.03) were independent predictors of OSA in patients with hypertension (Table 3).

### 3.4. Construction and Assessment of the Nomogram

**Figure 2:**
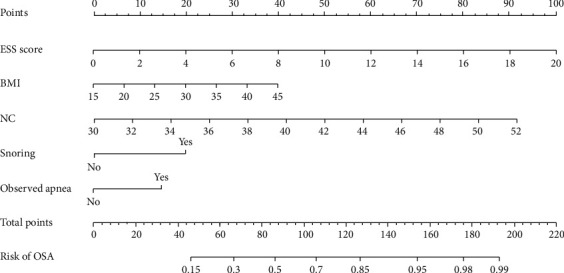
Proposed nomogram for OSA prediction (e.g., a resident with ESS = 10, BMI = 30, neck circumference = 42, snoring, no observed apnea, the total point is 140 read from the above nomogram, and the corresponding risk of OSA is 0.90 [90%]). OSA, obstructive sleep apnea; BMI, body mass index; ESS, Epworth sleepiness scale; NC, neck circumference.

Based on the results of multivariate logistic regression analysis, we constructed a nomogram to predict OSA (Figure 2), which can also be calculated using the formula: probability (OSA) = 1/(1 + exp (−(−9.980 + 0.216 × ESS score + 0.057 × BMI (kg/m^2^) + 0.180 × NC (cm) + 0.857 snoring (yes = 1, no = 0) + 0.629 × observed apnea (yes = 1, no = 0).

**Figure 3:**
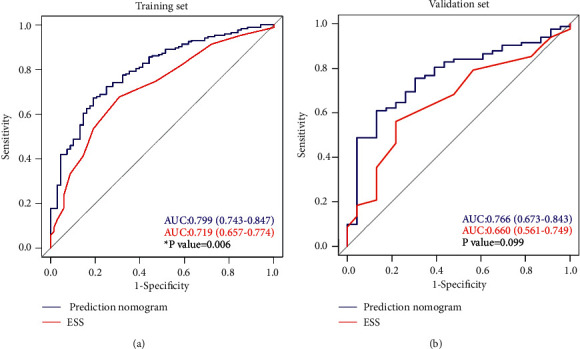
Receiver operating characteristic curve of the nomogram prediction and ESS in training set (a) and in validation set (b). The area under curve of the nomogram model is significantly larger than that of ESS. OSA, obstructive sleep apnea; ESS, Epworth sleepiness scale; AUC, area under the curve.

**Figure 4:**
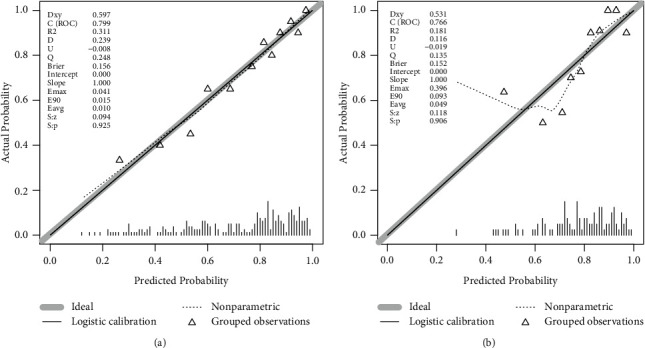
Calibration curves of the nomogram in training set (a) and in validation set (b). Notes: The *x*-axis represents the predicted OSA probability. The *y*-axis represents the actual identified patients with OSA. The diagonal dotted line represents a perfect prediction by an ideal model. The solid line represents the performance of the nomogram, of which a closer fit to the diagonal dotted line represents a better prediction. OSA, obstructive sleep apnea.

The AUC for the nomogram was 0.799 (95% CI: 0.743–0.847), indicating good discrimination (Figure 3(a)). The calibration curve plot indicated good agreement between the predicted and actual probability of OSA (*P*=0.925) (Figure 4(a)).

### 3.5. Internal Validation of the Nomogram

The nomogram showed good discrimination (AUC: 0.766; 95% CI: 0.673–0.843) (Figure 3(b)) and good calibration (*P*=0.906; Figure 4(b)) in the validation set. Thus, the nomogram performed well in the training and validation sets.

### 3.6. Clinical Value of the Nomogram

ROC analysis showed that the AUC of the nomogram was 0.799, significantly higher than that of the ESS (0.719) (*P*=0.006) (Figure 3(a)). The accuracy of the nomogram and the ESS was compared using the NRI and IDI. The NRI and IDI were 0.552 (95% CI: 0.282–0.822, *P* < 0.001) and 0.088 (95% CI: 0.045–0.133, *P* < 0.001), respectively (Table 4). These results indicated that the predictive accuracy of the nomogram was higher than that of the ESS.

**Figure 5:**
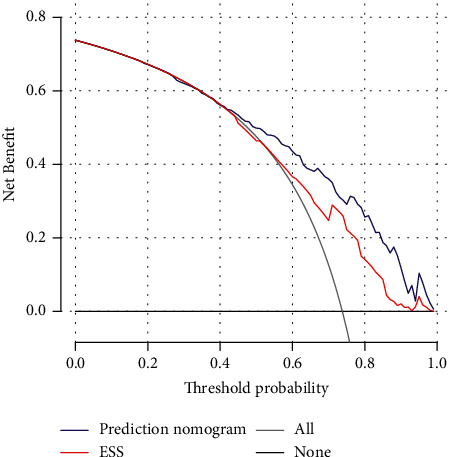
Decision curve analysis for the nomogram for predict OSA in validation set. Notes: The *y*-axis measures the net benefit. The blue line represents the OSA risk nomogram. The red line represents the ESS prediction risk of OSA. The thin solid line represents the assumption that all patients are non-OSA. Thin thick solid line represents the assumption that no patients are non-OSA. The decision curve showed that if the threshold probability of a patient and a doctor is more than 42%, using this nomogram for OSA in the current study to predict OSA risk adds more benefit than the ESS, intervention-all-patients scheme, or the intervention-none scheme. OSA, obstructive sleep apnea; ESS, Epworth sleepiness scale.

The clinical benefits of the nomogram and ESS were compared. DCA curves showed that the nomogram could better predict OSA and was more clinically beneficial than ESS at threshold probabilities of >42% using the treat-all-patients scheme and the treat-none scheme (Figure 5).

## 4. Discussion

This study developed and validated an easy-to-use nomogram model for diagnosing OSA. To our knowledge, this study is the first to establish a prediction model based on ESS scores and other OSA-related clinical parameters to distinguish OSA from non-OSA in patients with hypertension. The nomogram model demonstrated good accuracy and discrimination. Additionally, we assessed the quality of the nomogram using decision analysis (net benefit and decision curves) and measures related to reclassification tables (NRI and IDI).

Although the morbidity of OSA is high, the actual situation may be more serious. Young et al. reported that approximately 82% of men and 93% of women with moderate to severe OSA were not clinically diagnosed [29, 30]. In addition, a Sleep Heart Health study found that only 1.6% of patients with OSA were clinically diagnosed, and only 0.6% were treated [31]. These results indicate that the diagnosis and treatment of OSA are inadequate. The major reason for underdiagnosis is limitations in diagnostic methods. The gold standard is PSG; however, the equipment is expensive and requires specialized venues and analysts.

Biomarkers, scales, and models used to diagnose OSA have limitations. For instance, NC underestimates OSA in lean individuals [32], and the diagnostic accuracy of NC varies between races and ethnicities. In addition, the optimal cutoff values of NC for predicting OSA in non-Asian populations are inconsistent [33–35], and few studies have measured these values in Asian populations. Therefore, the optimal cutoff of NC for OSA diagnosis should be determined in different ethnic populations to improve the diagnostic performance of NC. In the present study, NC was considered as a continuous variable in the nomogram, combined with other OSA-related clinical measurements, increasing the predictive accuracy of NC in Asian patients with hypertension. The AUC of a nomogram model with eight predictors of OSA in Chinese adults was 0.84 [36]; however, the model was based on blood markers, which involved invasive tests, and did not include patients with hypertension. Aaronson et al. developed a prediction model of OSA by logistic regression analysis using sociodemographic factors, self-reported symptoms, and clinical features [37]; nonetheless, the study population consisted of patients with stroke, limiting the generalizability of the results.

OSA is screened using well-designed questionnaires, including the ESS and STOP-Bang. However, the results were not satisfactory [15]. For instance, Sil et al. [38] found that the AUC of the ESS was 0.672, indicating that this model was marginally useful for predicting OSA. The diagnostic sensitivity and specificity of the ESS for OSA were 54% and 65%, respectively [39], demonstrating the low accuracy of this scale to screen OSA. In the present study, DCA, NRI, and IDI indicated that at a probability threshold of >42%, the nomogram was more clinically beneficial and could better predict OSA than the ESS. STOP-Bang was initially used to screen surgical patients [40], and its ability to predict mild, moderate, and severe OSA was higher than that of the sleep apnea clinical score, BQ, and ESS [41]. However, the AUC of OSA for all degrees of severity (0.72) was lower than that of our model [20]. Additionally, although the SBQ seems more accurate than other questionnaires for screening OSA, its relatively low specificity limits its clinical utility [39]. Conversely, the ability of our nomogram to discriminate between OSA and non-OSA was high. Twelve potential risk predictors, including ESS- and OSA-related parameters, were used to construct the nomogram, and five predictors were selected using LASSO regression to reduce overfitting [42]. DCA weighs the benefits and risks by comparing the net benefits of models with different threshold probabilities versus performing PM examinations for all patients [43]. The results showed that our nomogram was more beneficial than treating all patients or treating none. Therefore, this model may improve decision-making and the early treatment of high-risk patients, particularly in clinics lacking PSG systems.

The proposed model detects OSA using clinical characteristics and ESS scores and can be easily applied in clinical settings by generating graphs of each case. Furthermore, this nomogram is noninvasive and uses objective and subjective parameters.

This study has limitations. First, our model was not externally validated in different ethnic groups and populations. Second, the single-center nature of the study may limit the generalizability of our findings. Third, using PM rather than standard PSG may have underestimated the severity of sleep apnea. However, PM is recommended by clinical guidelines for diagnosing OSA [18]. In addition, PM has unique advantages because sleep monitoring in a home environment is less likely to interfere with the routines of daily life, including sleep, and is better related to real sleep-disordered breathing in some cases [44].

## 5. Conclusions

We developed and validated an accurate nomogram model comprising the ESS and other OSA-related parameters to predict OSA in subjects with hypertension, improving clinical decision-making.

## Figures and Tables

**Table 1:** Characteristics of the subjects by validation set and training set.

	Training set (*n* = 242)	Validation set (*n* = 105)	Overall (347)	t/*χ*/*z*	*P-value*
Age, year	46.60 ± 12.33	46.67 ± 12.67	46.6 ± 12.4	0.046	0.963^a^
Sex, men (*n*, %)	182 (75.2)	88 (83.8)	270 (77.8)	3.139	0.091^b^
Occupation (*n*, %)					
Physical	82 (33.9)	30 (28.6)	112 (32.3)	3.327	0.189^b^
Office work	79 (32.6)	45 (42.9)	124 (35.7)		
Unclassifiable	81 (33.5)	30 (28.6)	111 (32.0)		
Height (cm)	169.1 ± 7.6	168.9 ± 6.9	169.1 ± 7.4	0.291	0.763^a^
Weight (kg)	79.4 ± 13.4	78.2 ± 12.6	79.0 ± 13.2	0.819	0.413^a^
BMI (kg/m^2^)	27.7 ± 3.7	27.3 ± 3.4	27.6 ± 3.6	0.873	0.383^a^
ESS score	6.5 ± 3.8	6.3 ± 3.8	6.4 ± 3.8	0.409	0.683^a^
AHI	13.4 [3.7–33.1]	10.8 [5.1–33.0]	12.1 [4.1–33.0]	0.065	0.948^c^
Snoring (*n*, %)	214 (88.4)	91 (86.7)	305 (87.9)	0.214	0.720^b^
Observed apnea (n, %)	84 (34.7)	39 (37.1)	123 (35.4)	0.189	0.714^b^
Tiredness (*n*, %)	138 (57.0)	62 (59.0)	200 (57.6)	0.726	0.813^b^
NC (cm)	40.6 ± 3.3	40.4 ± 2.8	40.5 ± 3.1	0.283	0.778^a^

OSA: Obstructive sleep apnea; BMI: body mass index; ESS: Epworth sleepiness scale; AHI: apnea-hypopnea index; NC: neck circumference. Mean ± SD, median (25th and 75th percentiles), or N (column %). *P* values: a = *t*-test, b = Pearson's chi-square test, and c = Wilcoxon rank-sum test.

**Table 2:** Predictors of OSA according to the LASSO regression model in patients with hypertension.

Predictors	Coefficients	Lambda.1se
ESS (per 1 score)	0.09208777	0.05989607
BMI	0.01032786	
NC	0.05860992	
Snoring	0.18728668	
Observed apnea	0.05838322	

OSA, obstructive sleep apnea; LASSO, least absolute shrinkage and selection operator; ESS, Epworth sleepiness scale; BMI, body mass index; NC, neck circumference.

**Table 3:** Prediction factors for OSA from study population by multiple logistic regression analysis.

Stratification	Β	OR (95%CI)	*P*-value
ESS (per 1 score)	0.216	1.24 (1.12–1.38)	<0.001
BMI	0.057	1.06 (0.94–1.20)	0.353
NC	0.180	1.20 (1.04–1.38)	0.013
Snoring	0.857	2.36 (0.94–5.92)	0.068
Observed apnea	0.629	1.88 (0.87–4.03)	0.107
Intercept	−9.980	—	<0.001

OSA, Obstructive sleep apnea; ESS, Epworth sleepiness scale; BMI, Body mass index; NC, neck circumference; OR, odds ratio; CI, confidence interval.

**Table 4:** Reclassification analyses for ESS and prediction nomogram to improve the risk prediction of OSA.

	Category-free NRI		IDI	
	Estimate (95% CI)	*P*-value	Estimate (95% CI)	*P*-value
ESS	Reference	—	Reference	—
Prediction nomogram	0.552 (0.282–0.822)	<0.001	0.088 (0.045–0.133)	<0.001

Notes: prediction nomogram included ESS, BMI, neck circumference, snoring, and observed apnea. OSA, obstructive sleep apnea; BMI, body mass index; ESS, Epworth sleepiness scale; IDI, integrated discrimination improvement; NRI, net reclassification index; CI, confidence interval.

## Data Availability

Materials included in the article, including all relevant raw data, will be made freely available to any researchers who wish to use them for noncommercial purposes, while preserving any necessary confidentiality and anonymity.
